# The impact of unethical pro-organizational behavior on employee well-being

**DOI:** 10.1038/s41598-026-49742-2

**Published:** 2026-04-20

**Authors:** Jing Wang, Qing Zhou, Miao Wang, Xu Yan

**Affiliations:** School of Management, Zunyi Medical University, Zunyi, China

**Keywords:** Initiative UPB, compulsory UPB, Basic psychological needs, Workplace well-being, Health occupations, Psychology, Psychology

## Abstract

Unethical pro-organizational behavior (UPB) has produced inconsistent effects on employee workplace well-being. Past studies often treat UPB as a unidimensional construct and, hence fail to consider its heterogeneous motivational characteristics. Based on self-determination theory, we examine the separate effects of two distinct UPB subtypes—IUPB and CUPB—on workplace well-being, with basic psychological needs satisfaction playing a mediating role. Data were collected from Chinese salespeople at three time points and 207 valid participants. The hypotheses were tested by confirmatory factor analysis, hierarchical regression, and bootstrap mediation analysis. Results show that CUPB negatively predicts workplace well-being, while IUPB does not have such an effect. The negative effect of CUPB on well-being is fully mediated by basic psychological needs satisfaction, with different roles played by the basic psychological needs satisfaction of autonomy, competence, and relatedness. This study adds to the literature by assessing the multidimensional structure of UPB and shedding light on the motivational driving forces that underlie its opposite effects on employee well-being. The findings also provide guidance to organizations in terms of reducing compelled unethical behavior and enhancing employee well-being through protecting basic psychological needs and moral workplace climates.

## Introduction

Unethical pro-organizational behavior (UPB)—that is, employees’ unethical behaviors aimed at benefiting their organization and organization members—is receiving tremendous attention in organizational behavior because it is located at the nexus of organizational performance pressure and ethical standards^1^. UPB can provide short-term benefits to the organization (for instance, safeguarding the firm’s image or improving sales profits); yet, it can also influence employees’ psychological functioning in ways that aggregate over time. If UPB systematically damages well-being, then organizations may be succeeding at the cost of hidden human and sustainability risks; if UPB does not, then scholars need to explain why unethical behaviour can sometimes occur without showing psychological discomfort for the actor. Yet, extant findings about whether UPB harm or benefit employees are mixed; theory and practice have no clue as to what the answer is. This inconsistency has been identified as one of the key unsolved gaps in recent systematic reviews of research on UPB^2,3^, which highlight the need for empirical studies on the multidimensional nature of UPB to integrate current mixed findings. The pressing need for this investigation is yet further increased by current workplace realities: post-pandemic organisations face tremendous performance pressures^4,5^. Therefore, many others have followed, generating UPB explosions in turn, and leading to a flurry of UPB enactments, while employee mental well-being has now become an essential organization sustainability indicator^6^. Filling this gap is now timely, as it can transform academic knowledge into strong interventions to balance organizational and employee mental well-being—a call to action for both researchers and practitioners.

So, to make progress, researchers need to move away from treating UPB as a whole construct and rather uncover which aspects of UPB lead to harmful outcomes versus benign outcomes. One promising route to do this is to distinguish different types of UPB based on the motivational and volitional conditions under which different UPBs occur, and then test the psychological processes linking each form to well-being. Without such a finer-grained approach, research findings will vary, according to which form of UPB happens to be predominant in a sample or situation—something that has been emphasized in recent calls for subtype-based UPB research^7,8^.

But inconsistency in the past evidence tends to come down to UPB’s “paradoxical nature,” while an underexplored explanation is that UPB may be multidimensional. Studies focusing on negative effects suggest that unethical acts evoke guilt, shame, stress, and emotional ambivalence^9–11^, whereas other studies have suggested potential positive effects, such as enhanced organization-based self-esteem, positive emotions, gains in resources^5,12^. These two sets of conclusions don’t sound contradictory to each other if the UPB measured across studies mean something different from each other. More specifically, research suggested that UPB can be disentangled into initiative UPB (IUPB) and enacted voluntarily to help the organization, and opposed to the compulsory UPB (CUPB), enacted under external pressure (for example, demand of a supervisor, intense performance pressure)^13^. Moreover, if employees are willing to engage in UPB (versus unwillingly), they should experience different psychological states and thus, different levels of workplace well-being. But, although this distinction makes a difference for explaining mixed findings, theoretical significance notwithstanding, very little research examined whether IUPB and CUPB have heterogeneous effects on workplace well-being or even clarified the causal mechanism. Two recent meta-analyses highlight the substantial heterogeneity in UPB’s consequences and motivational antecedents, yet reveal that existing research continues to treat UPB as a homogeneous construct^8,14^. Most existing research do not test for the multidimensional view of UPB that distinguishes initiative versus compulsory UPB and integrates self-determination theory to examine their differential effects on workplace well-being: these gaps still remain.

But this gap remains partly because much of the UPB literature implicitly sidesteps a key issue: voluntariness. In collapsing all UPB into a single score, prior research also missed that “unethical behavior for the organization” can be experienced as both self-endorsed action and as forced compliance. This is important because the same outward behavior (for example, misrepresenting information to clients) can have very different psychological meanings. When enacted under pressure, employees might feel they are acting against their values, giving up control over their own decisions, and putting themselves at risk of blame if the wrongdoing is discovered—in circumstances that are truly beneficial to well-being. By explicitly critiquing this unidimensional treatment of UPB, our present study aims to explain why UPB relates to well-being but prior evidence appears inconsistent across studies that probably were a mix of voluntary and pressured unethical enactment. Significantly, this oversight has been noted in some recent critiques of the UPB methodology^15–17^, that note, rather alarmingly, that ignoring voluntariness means that aggregate findings from UPB are not meaningful.

Existing research provides important groundwork and also reveals the need for this refinement. UPB was initially conceived of as unethical acts performed in the name of the firm, shaped by factors such as organizational identification and reciprocity beliefs^1,18^. Subsequent research also included antecedents and outcomes, harmful consequences for UPB actors (e.g., guilt and ambivalence^10^; strain and conflict^11^), as well as beneficial or mixed outcomes (e.g., self-esteem or resource-related gains)^3,12^. More recently, one study advanced the field by reconceptualizing UPB as comprising IUPB and CUPB and developing measures accordingly^13^. Yet this subtype perspective has not been sufficiently integrated with a theoretically grounded account of how UPB translates into employee well-being, leaving the psychological pathway that should differentiate IUPB from CUPB insufficiently tested. As noted in recent review of organizational ethics research^19,20^, the field lacks targeted theoretical frameworks that link UPB’s motivational bases to employee psychological outcomes—a gap the present study aims to fill.

The present study extends prior research by combining the subtype distinction with self-determination theory (SDT) to specify a mechanism that is directly relevant to the voluntariness critique. SDT posits that autonomy, competence, and relatedness are basic psychological needs essential for well-being; satisfaction of these needs enhances well-being, whereas need frustration undermines it^21–23^. This framework is particularly suitable for distinguishing UPB subtypes because the defining feature separating IUPB from CUPB is precisely the degree of volition versus external pressure— a distinction that aligns with SDT’s core focus on motivational antecedents of well-being^24,25^.Unlike prior studies that rely on general theories (e.g., Conservation of Resources theory) to explain UPB’s effects^11,12,26^, SDT provides a targeted lens to unpack why coerced versus voluntary unethical behavior would shape well-being differently. CUPB, by definition, should thwart autonomy and may also undermine relatedness and competence through moral conflict, strained relationships, and self-doubt, thereby reducing workplace well-being. In contrast, IUPB is enacted with greater volition, which may lessen autonomy loss and produce a more ambiguous overall effect on well-being. In this way, the current study differs from earlier work that either (a) treated UPB as unidimensional or (b) discussed mixed outcomes without directly testing whether pressure-driven UPB is the primary source of well-being impairment.

Accordingly, this study pursues three objectives: (1) to empirically validate UPB’s multidimensionality by distinguishing IUPB and CUPB; (2) to examine whether IUPB and CUPB are differentially associated with employees’ workplace well-being; and (3) to test whether basic psychological needs satisfaction (autonomy, competence, and relatedness) mediates the relationship between CUPB and workplace well-being.

By doing so, the paper contributes to the literature in three ways. First, it helps reconcile inconsistent findings on UPB’s consequences by showing that well-being outcomes depend on the subtype of UPB being examined rather than on UPB in general—addressing a key call in recent UPB reviews^2,3^. Second, it introduces an SDT-based explanatory pathway—basic psychological needs satisfaction—that clarifies why compelled unethical behavior should be particularly harmful to employees, advancing the field’s understanding of UPB’s psychological mechanisms^25^. Third, it encourages a shift in UPB research from asking whether UPB is “good or bad” for the actor to identifying which forms of UPB are most psychologically costly, thereby offering a more precise foundation for subsequent theorizing and empirical investigation—aligning with the latest trends in organizational ethics research^6^.

## Theoretical background and hypotheses

### Self-determination theory

Self-Determination Theory (SDT)^21^ proposes that human beings have three innate psychological needs—autonomy, competence, and relatedness—and that meeting those needs is important for optimal functioning, intrinsic motivation, and psychological well-being. Autonomy means the need to act volitionally and according to one’s values; competence refers to the need to be effective in one’s environment; and relatedness refers to the need to be connected to and cared about by others. In SDT, autonomous motivation is distinguished from controlled motivation, that is, the act of performing to satisfy external pressure rather than genuine interest or personal endorsement. In summary, autonomous motivation tends to satisfy basic psychological needs and promote well-being, whereas controlled motivation diminishes psychological needs and consequently well-being^22,27^.

SDT has particular advantages in distinguishing IUPB from CUPB, since the distinction that defines these two subtypes is precisely the degree of volition versus external pressure—a distinction that exactly maps onto the heart of what SDT is concerned about: motivational quality. IUPB, being voluntary and intrinsic, translates into autonomous motivation; CUPB, being compelled by external demands, translates into controlled motivation. This conceptual fit means that SDT can explain not only why these two subtypes may produce different effects on well-being but also how these effects might occur—through the mediating factor of satisfaction of basic needs.

### Conservation of resources theory

Despite having provided the primary explanation of the mediating processes, COR theory^28^ complements SDT by providing an explanation of the direct effects of the UPB subtypes on workplace well-being. COR theory posits that individuals strive to acquire, protect, and retain valued resources (e.g., self-esteem, social support, energy), and that it is stressful when resources are lost or threatened. Workplace well-being indicates the balance of the net resource gains and resource losses caused by work behaviors.

### Initiative UPB and compulsory UPB

UPB is a common phenomenon at work, and has attracted interests of scholars and practitioners alike. Most of the existing research work focuses on identifying the antecedents of UPB (e.g., organizational identification, leadership styles) but rarely discusses the consequences of UPB for the employees who exhibit this behavior^2^. However, some work has emerged recently studying these consequences^2,10–12,29^. A key limitation of the earlier work is that UPB has been assumed as a unidimensional construct and each form of UPB is regarded as the same entity. Some scholars have challenged the assumption of a unidimensional construct of UPB by distinguishing between two subtypes^13^:

Initiative UPB (IUPB) is an unethical behavior that is performed voluntarily by employees for the organization, driven by intrinsic motivations such as a desire to contribute to organizational success. For example, an employee might proactively misrepresent information to secure a contract for the company, believing this action aligns with their own goals and values. In contrast, Compulsory UPB (CUPB) are unethical behaviors with which employees agree because of strong external pressures, such as demands from supervisors, organizational expectations, or production goals and so on. For example, an employee may feel compelled to withhold negative information from clients because their manager asked them to do so, even if they themselves might be against it.

These two forms of UPB have distinct motivational bases (intrinsic vs. extrinsic), and are therefore likely to be associated with different psychological and behavioral effects for actors^13^. This distinction helps clarify the mixed findings in prior research.

### UPB and workplace well-being

#### The relationship between IUPB and workplace well-being

From the COR theory perspective, IUPB should enhance workplace well-being because it should lead to net resource gains. First, IUPB is voluntary and in line with the actor’s intrinsic motives, making it less likely to lead to depletion of resources^30^. Autonomy—a basic human need— is satisfied when people act in volition and satisfying autonomy needs is associated with better well-being^22^. Second, engaging in IUPB may help employees meet their personal goals (e.g., gaining recognition, contributing to the organization), thereby accumulating personal resources such as self-esteem and organizational-based self-worth^12^. For instance, employees who proactively engage in UPB may develop a sense of being a valuable member of the organization, which can improve their self-evaluation and sense of effectiveness^3,21,31^.

However, IUPB is still an unethical behavior, and its inherent unethicality may elicit negative consequences that deplete resources. For example, employees who engage in IUPB may experience emotional ambivalence^10^ due to the problematic tension between their desire to benefit the organization and their awareness that they are breaking social norm rules. Moreover, individual employees could feel guilt or shame after engaging in unethical behavior (despite voluntary behavior) because they recognize that they are acting contrary to a broader societal moral standard^32^. Such negative emotions may deplete psychological resources, and compromise their workplace well-being. Given these competing possibilities, we propose two opposing hypotheses:

##### Hypothesis 1a (H1a)

Initiative UPB (IUPB) is positively related to employees’ workplace well-being.

##### Hypothesis 1b (H1b)

Initiative UPB (IUPB) is negatively related to employees’ workplace well-being.

#### The relationship between CUPB and workplace well-being

In contrast, CUPB tends to decrease workplace well-being because of net resource depletion. According to COR theory, acting under external pressures tends to deplete resources because these acts do not flow from the actor’s motives. CUPB, being compelled by externalities deprives actors of autonomy they need, and therefore causes psychological distress^30^. CUPB can also foster negative affect such as anxiety, guilt or resentment, which deplete affective resources^9,29^.

Second, CUPB also exposes employees to risks (e.g., being held responsible for unethical actions if caught. Fear of negative outcomes (e.g., reputation damage, disciplinary action) is cognitively and emotionally exhausting to deal with and this is a use of resources^33^. Over time, such resource depletion would cause loss of workplace well-being. Thus, we hypothesize:

##### Hypothesis 2 (H2)

Compulsory UPB (CUPB) is negatively related to employees’ workplace well-being.

### UPB and basic psychological needs satisfaction

To further explore the mechanisms underlying the relationship between UPB and workplace well-being, we integrate Self-Determination Theory (SDT)^21^. SDT provides three basic psychological needs that are universal and are essential for human growth, integrity, and well-being:

***Autonomy:*** The need to be in control of one’s own behavior, to be internally guided, and to act in line with one’s own values.

***Competence:*** The need to feel competent in responding to one’s environment and to feel effective at achieving desired goals.

***Relatedness:*** The need to feel connected to other people, to be cared for, and to care for others.

SDT specifies that satisfaction of these needs is essential for psychological health and well-being and their frustration leads to maladjustment. Behaviors fueled by intrinsic motivation tend to satisfy these needs, and behaviors fueled by extrinsic motivation (i.e., behaviors motivated by external pressures) are more likely to frustrate them^27^.

#### The relationship between IUPB and basic psychological needs satisfaction

Because IUPB is voluntary and driven by intrinsic motivation, IUPB is expected to enhance satisfaction of basic psychological needs. IUPB is done voluntarily, so doing IUPB satisfies the need for autonomy^31^. Besides, IUPB is usually perceived as a “heroic sacrifice” for the organization and this may engender positive feelings such as pride^10^ and an element of accomplishment^34^. Thus, IUPB enhances employees’ sense of competence because they feel that they have made a useful contribution to organizational success. IUPB benefits both the organization and its members, and this may evoke appreciation from supervisors and co-workers^35,36^. Such positive social feedback makes employees feel socially connected and satisfy the need for relatedness. Thus, we propose:

##### Hypothesis 3a (H3a)

Initiative UPB (IUPB) is positively related to the satisfaction of basic psychological needs (autonomy, competence, and relatedness).

#### The relationship between CUPB and basic psychological needs satisfaction

Because CUPB is supported by external pressure but not intrinsic motivation, it is likely to frustrate basic psychological needs. CUPB is coerced, violating the need for autonomy. Employees engaging in CUPB do not behave in line with their own values, creating a feeling of helplessness and lower autonomy satisfaction^37^. CUPB can involve behaviour that violates employees’ own personal ethics, creating conflicts in beliefs and doubts about themselves^9^. This will therefore negatively affect their sense of competence, as they will be in doubt about how they can do things effectively while still acting consistent with their values. What is more, CUPB may damage working relationships. Employees might feel isolated from colleagues who disapprove of their unethical behavior or might hate their boss for forcing them into CUPB^38^. Relatedness satisfaction is lowered. Thus, we hypothesize:

##### Hypothesis 3b (H3b)

Compulsory UPB (CUPB) is negatively related to the satisfaction of basic psychological needs (autonomy, competence, and relatedness).

### The mediating role of basic psychological needs satisfaction

SDT emphasizes that basic psychological needs satisfaction is a key mediator between environmental factors (e.g., work behaviors) and well-being^21^. Prior studies have shown that basic psychological needs satisfaction mediates the relationship between various work factors (e.g., leadership, job characteristics) and outcomes such as job satisfaction and life satisfaction^38–42^.

We propose that basic psychological needs satisfaction mediates the relationship between UPB and workplace well-being. Specifically:

For CUPB: As hypothesized, CUPB frustrates basic psychological needs (H3b). Since these needs are essential for well-being, their frustration would directly reduce workplace well-being. Thus, the negative effect of CUPB on workplace well-being is likely to operate through reduced satisfaction of autonomy, competence, and relatedness needs.

For IUPB: If IUPB enhances basic psychological needs satisfaction (H3a), this satisfaction would, in turn, improve workplace well-being. However, given the competing hypotheses about IUPB’s direct effect on well-being (H1a and H1b), the mediating role of needs satisfaction for IUPB is less clear and will be explored empirically.

Given the stronger theoretical basis for CUPB’s effects, we focus on hypothesizing the mediating role for CUPB:

#### Hypothesis 4 (H4)

The satisfaction of basic psychological needs (autonomy, competence, and relatedness) mediates the negative relationship between CUPB and workplace well-being.

### Integrated conceptual model

**Fig. 1:**
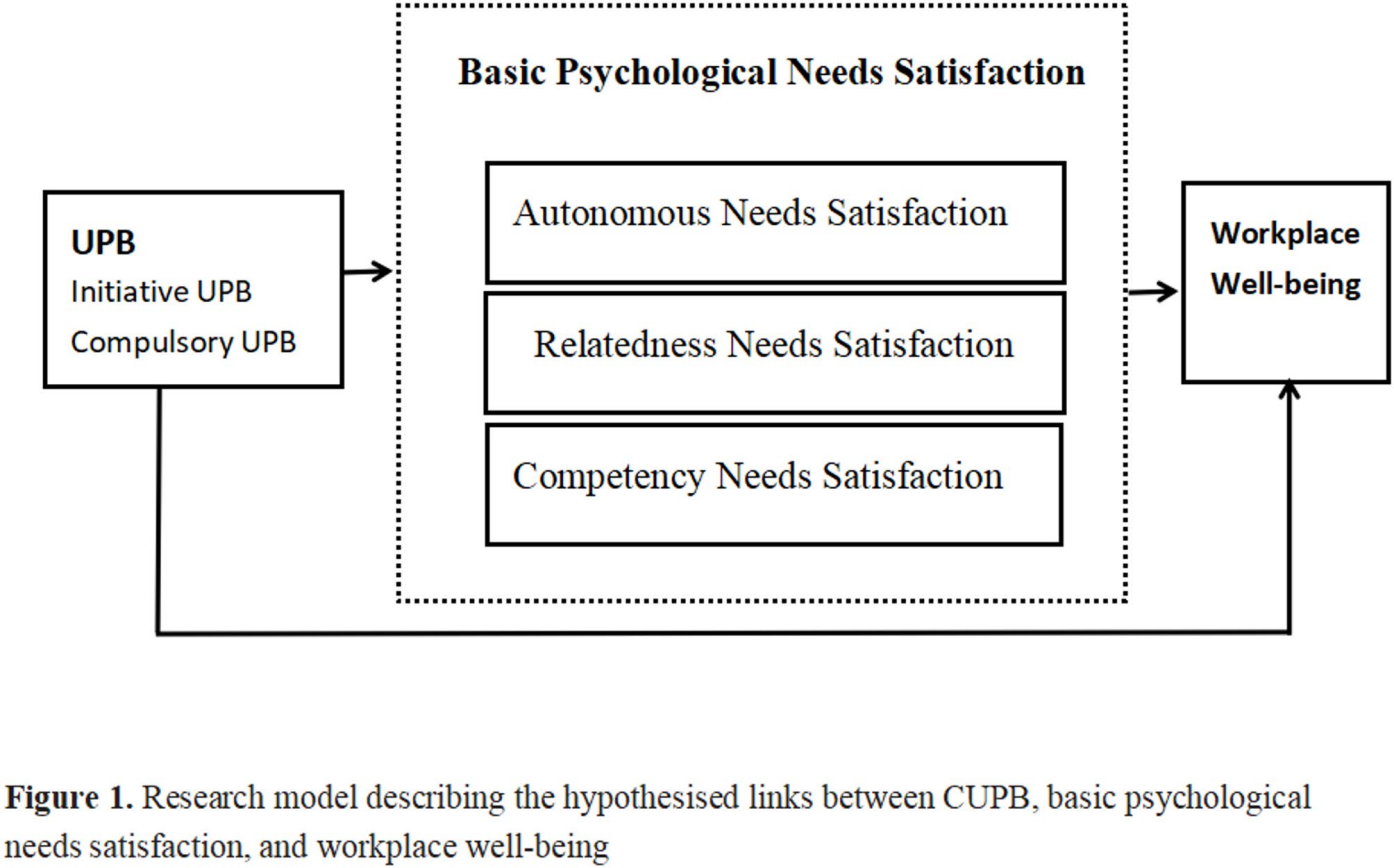
Research model describing the hypothesized links between UPB, basic psychological needs satisfaction, and workplace well-being.

Figure 1 integrates the theoretical perspectives and hypothesized relationships. Our model proposes that CUPB negatively impacts workplace well-being both directly (via resource depletion, as in COR theory) and indirectly (via frustration of basic psychological needs, as in SDT). IUPB’s effects are treated as exploratory because of the competing theoretical influence. Basic psychological needs satisfaction serves as the core mediating mechanism linking CUPB to well-being.

## Method

The methodological design for our study is grounded in self-determination theory (SDT) and conservation of resources (COR) theory. Methods were chosen to align with our research goals and theoretical framework. The three-wave data collection design addresses common method bias^43^ and establishes the temporal order of variables—UPB subtypes leading to basic psychological needs satisfaction and then workplace well-being—which is consistent with the hypothesized causal relationships. Our choice of established scales guarantees measurement validity and the sampling strategy as well as the control variables enhance the study’s internal and external validity.

### Research procedure and sample

#### Sampling technique and rationale

In this study, we used purposive sampling as well as snowball sampling. Salespeople were purposively sampled as the sample because they often experience high performance pressure, such as sales targets, customers’ needs. They are also more likely to encounter situations demanding trade-offs between ethics and organization interests^1,13^. It makes the sample relevant to the research topic because the UPB phenomenon is more salient in this group of people, which can help to fully capture the variance of the key variables IUPB and CUPB.

For unethical behaviors such as UPB, direct recruit of participants through the public can result in very low response rate or social desirability bias. We recruited the first batch of participants through professional contacts, such as university alumni doing sales and corporate HR counterparts. These initial participants were invited to recommend eligible colleagues who meet the selection criteria outlined earlier in this study. Snowballing thus facilitates trust in participants and willingness to respond truthfully, enhancing data quality^43^.

The sampling implementation process unfolded as follows. First, 30 initial participants were collected by purposive sampling, all meeting the selection criteria. Second, each initial participant was encouraged to recommend 2–3 colleagues working in sales with at least 1 year of work experience. Third, all recommended participants were screened against the selection criteria to ensure their eligibility. Finally, we removed the duplicates or ineligible participants and had 207 final valid sample participants.

#### Participant selection criteria and justification

Participants were selected based on four key criteria with corresponding theoretical and practical justifications. First, their occupation was limited to full-time salespeople. As stated earlier in this study, salespeople face inherent performance pressure and frequent ethical dilemmas of UPB such as misrepresenting product information to meet targets, making them an appropriate sample to test the hypothesized relationships^10,11^.

Second, participants needed at least 1 year of work experience in their current job. Less than 1 year of work experience would mean that employees had few occurrences requiring UPB or formed adequate perceptions about the benefits of workplace well-being, making it hard to produce reliable responses^29^.

Third, participation was voluntary and complete all three waves of surveys. Voluntary participation means that participants are motivated to give honest answers to surveys and therefore do not have a response bias^43^.

Fourth, participants answered the attention-check items correctly on each wave of the surveys (e.g., “Please choose ‘strongly agree’ for this item.”). This criterion helps exclude careless respondents and ensures data quality^44,45^.

#### Data collection procedure

Data were collected via Wenjuanxing, an online survey platform widely used in academic research in China^29^. Prior to participation, potential participants read a consent form detailing the study’s purpose, the voluntary nature of involvement, confidentiality of responses and the right to withdraw at any time. The study protocol was reviewed and approved by the Ethics Committee of Zunyi Medical University (NO. [2022] 2–015), and all procedures complied with relevant ethical standards for research involving human participants. All participants provided informed consent prior to participation.

Participants completed three surveys at one-week intervals to reduce common method bias^43^. Time 1 focused on measures of IUPB and CUPB. Time 2 included measures of basic psychological needs satisfaction, control variables, including age, gender, education level, organizational tenure and job position, as well as attention-check items. Time 3 centered on the measure of workplace well-being and additional attention-check items.

A total of 220 participants initiated the survey and 207 completed all three waves, resulting in a response rate of 94.1%. Participants who failed attention-check items (n = 13) were excluded from the final analysis to ensure data quality.

#### Sample characteristics

The final sample consisted of 207 Chinese salespeople from various organizations across the sales and service industries. Demographically, 45.4% were female and 54.6% were male. Age distribution included 25–30 years old (42.5%), 31–40 years old (38.2%) and above 40 years old (19.3). Education levels included associate degree (18.3%), bachelor’s degree (73.6%), and master’s degree or above (8.1%); no participants had a high school education or below. Organizational tenure breakdown included 1–4 years (35.2%), 5–8 years (47.6%) and above 8 years (17.2%).

Organizational types represented included private enterprises (50.5%), state-owned enterprises (31.4%) and foreign-funded enterprises (18.1%). Industry distribution included retail (32.4%), technology (28.6%), finance (21.3%) and service (17.7%).

Salespeople were recruited because they often face high performance pressure and are more likely to engage in UPB, such as misrepresenting product information to meet targets, making them a relevant sample for this study.

#### Sample generalizability and representativeness

Our target population of the study is Chinese employees under performance pressure and may engage in UPB, and among them a target group was salespeople, an occupation that is closely related to our research context. In order to make the sample more representative, we adopted a purposive sampling and snowball sampling methods to select participants from different backgrounds.

Industry diversity was achieved by having the participants from different industries (such as retail, technology, finance and service). Organizational type diversity was achieved by having employees from private enterprise, state-owned enterprise and foreign-funded enterprise, covering different management type and ethical climate context of organizations.

The demographic composition of the sample in terms of gender, age distribution, educational attainment, and organizational tenure generally corresponds with broader profiles of China’s working population reported in national human resources surveys (e.g., China Human Capital Report 2024; China Human Resources Development Report 2024), which supports our sample as a representative portrait of the target population.

Despite these efforts, the sample here concerns Chinese salespeople only, limiting generalizations of our findings to occupations other than sales or to cultures other than Chinese. We discuss this limitation and call for future replicating research on cross-occupational and cross-cultural aspects in detail in the “Limitations and Future Directions” section.

#### Control variables and justification

Consistent with previous research^13,18^, we controlled for several demographic variables that may influence UPB enactment or workplace well-being. Age was controlled as it may be related to psychological resource accumulation and ethical reasoning^27^^.^ Gender was controlled as previous studies found evidence of this variable’s potential influence on one’s decision to act in an ethical manner and emotions in organizations^18^. Education level and organizational tenure were controlled as they might reflect individuals’ cognitive resources and social capital, as well as their exposure to organizational norms, all of which would affect their experienced UPB and well-being^29^. Job position was included because jobs that are more managerial in nature could mean a different level of autonomy and pressure experienced by workers which, in turn, could relate to UPB enactment and subsequent psychological outcomes^13^.

### Research instruments

All measures were adapted from established scales with established reliability and validity. Translation was carried out according to high standards and to make sure the content is culturally appropriate and measures are equivalent, a pilot test was conducted to refine the questionnaire.

#### Translation of questionnaires

As all the original scales developed in English were taken, they were translated into Chinese following the back-translation method^46^, which ensured the cultural appropriateness and meaning congruency of the instrument to Chinese respondents. Five procedures were conducted.

First, forward translation was completed by two bilingual researchers fluent in both English and Chinese with academic backgrounds in organizational behavior. They independently translated the original English scales into Chinese. The two translations of these original English scales were compared and discussed to resolve discrepancies, resulting in a preliminary Chinese version.

Second, back translation was performed by a third bilingual researcher blind to the original English scales. This researcher translated the preliminary Chinese version back into English.

Third, a consensus check was conducted. The research team compared the back-translated English version and the original English scales. Items with semantically inconsistent terms were found and rewritten such as changing the term “misrepresent the truth” to “offer misleading information” to fit the style of Chinese work-related language.

Fourth, cultural adaptation was carried out. The revised Chinese version was reviewed by three sales managers with more than 10 years’ work experience to ensure items were contextually appropriate for Chinese salespeople. For example, “supervisor demands” was changed to “supervisor requirements” to make it consistent with Chinese organizational communication norms.

Fifth, finalization was done after incorporating all modifications. The final Chinese version fully conveyed the meaning of each item of the original scale while being culturally relevant and easy to understand.

#### Key variables

*Initiative UPB (IUPB)*: At Time 1, IUPB was measured using a 6-item scale developed by^18^. Participants rated their degree of agreement for each item of the scale on a 7-point Likert scale ranging from 1 (strongly disagree) to 7 (strongly agree). A sample item was: “If it would help my organization, I would provide inaccurate information to make my organization look good.” In the current study, the Cronbach’s α for this scale was 0.79, indicating good internal consistency.

*Compulsory UPB (CUPB)*: At Time2, CUPB was measured using a 7-item scale^13^. Participants rated the frequency of each behavior on a 7-point scale (1 = never, 7 = more than once a day). A sample item was: “I had to provide inaccurate information to make my organization look good.” The Cronbach’s α for this scale in the current study was 0.85, indicating good internal consistency.

*Basic psychological needs satisfaction (BPN)*: At Time 2, basic psychological needs satisfaction was assessed using a 9-item scale^47^. The scale comprises three subscales. The autonomy subscale includes three items such as “I feel more autonomous in this job.” The competence subscale features three items including “I think I can do this job well.” The relatedness subscale has three reverse-coded items such as “I really don’t fit in with other people at work.” Participants rated their level of agreement with each item on a 6-point Likert scale ranging from 1 (“strongly disagree”) to 6 (“strongly agree”). The Cronbach’s α for the overall scale was 0.82, with α = 0.80 for the autonomy subscale, 0.78 for the competence subscale, and 0.76 for the relatedness subscale, all of which could achieve an acceptable consistency.

*Workplace well-being:* At Time 3, workplace well-being was measured using a 6-item scale^48^. Participants rated their agreement with each item on a 6-point Likert scale (1 = strongly disagree, 6 = strongly agree). Sample items include: “I feel happy when I am working” and “I am satisfied with my current work.” The Cronbach’s α for this scale in this study was 0.82, indicating good internal consistency.

#### Pilot test and pre-test explanation

A pilot test was conducted prior to the full-scale study to evaluate the reliability of the scales, clarity of questionnaire items, and cultural appropriateness of the Chinese translations.

*Pilot test sample*: The pilot sample was 50 full-time salespeople recruited from two enterprises in the retail and technology sectors (25 participants per industry), but we excluded the sample from the final study sample to avoid response bias. Participants also matched the same basic selection criteria in the formal study: at least 1 year of sales experience, voluntary participation, and willingness to complete the full questionnaire. Demographically, the pilot sample was also consistent with the target population: 48% female, 52% male; 40% aged 25–30 years, 36% aged 31–40 years, 24% aged above 40 years; 70% holding a bachelor’s degree, 22% with an associate degree, and 8% with a master’s degree or above.

*Implementation procedure*: Participants completed the preliminary Chinese version of the questionnaire (encompassing all key variables: IUPB, CUPB, basic psychological needs satisfaction, workplace well-being, and demographic items) via Wenjuanxing. After completing the survey, they were invited to give constructive feedback on: (1) the clarity of item wording (e.g., whether any expressions were ambiguous or difficult to understand); (2) the relevance of items to their daily sales work; and (3) the overall response burden (e.g., survey length, ease of completion). The pilot survey took about 10 min to finish, and we paid an extra small monetary incentive (¥20) for them.

*Data analysis and results*: Descriptive statistics and internal consistency reliability (Cronbach’s α) were calculated for each scale using SPSS 26.0. The results showed good reliability for all the scales, with Cronbach’s α exceeding the acceptable threshold of 0.70^49^ for all scales. Specifically, the reliability coefficients were 0.76 for initiative UPB, 0.88 for compulsory UPB, and 0.84 for workplace well-being scale. For basic psychological needs satisfaction, the overall scale demonstrated a Cronbach’s α of 0.85, with subscale α of 0.71 for autonomy, 0.83 for competence, and 0.74 for relatedness.

*Revisions based on feedback*: Open-ended feedback from pilot participants suggested two targeted revisions to improve item clarity and specific job context:

The IUPB item “If it would help my organization, I would misrepresent the truth to make my organization look good” was revised to “If it would help my organization, I would provide inaccurate information to make my organization look good.” Participants noted that “misrepresent the truth” felt overly formal and moralized in a workplace context, while “provide inaccurate information” better reflected the subtle ethical dilemmas they encounter in sales roles.

The autonomy subscale item “I feel more autonomous in this job” was modified to “I feel I have control over my work tasks.” Pilot participants indicated that “autonomous” was too abstract; the revised wording directly aligns with the practical experience of salespeople (e.g., controlling client communication, task scheduling).

No additional modifications were needed, as participants reported no other ambiguities, and the survey length was deemed appropriate. The revised version of the questionnaire from the pilot test was used for the full-scale survey to assure validity of the measures and data quality.

## Results

### Confirmatory factor analysis

To test the discriminant validity of the key variables (IUPB, CUPB, basic psychological needs satisfaction, workplace well-being), we conducted a confirmatory factor analyses (CFA) using Mplus 8.3 (Official URL: https://www.statmodel.com/), in which we compared our proposed four-factor model with several alternative models: the four-factor model (Model 1) treats IUPB, CUPB, basic psychological needs satisfaction, and workplace well-being as distinct factors; the three-factor model (Model 2) combines IUPB and CUPB into a single UPB factor while keeping the other two factors unchanged; the two-factor model (Model 3) merges UPB (IUPB + CUPB) and basic psychological needs satisfaction into one factor, with workplace well-being as the second factor; and the one-factor model (Model 4) loads all items onto a single factor. Model fit was evaluated using the following indices: χ^2^/df (ideal < 3), Comparative Fit Index (CFI; ideal > 0.90), Incremental Fit Index (IFI; ideal > 0.90), Standardized Root Mean Square Residual (SRMR; ideal < 0.08), and Root Mean Square Error of Approximation (RMSEA; ideal < 0.08). As shown in Table 1, the four-factor model provided the best fit to the data (χ^2^/df = 1.65, CFI = 0.94, IFI = 0.94, SRMR = 0.07, RMSEA = 0.06), significantly outperforming all alternative models. This confirms the discriminant validity of the key variables.

**Table 1:** Confirmatory factor analysis results.

Model	χ^2^	df	χ^2^ /df	CFI	IFI	SRMR	RMSEA
Four-factor model: IUPB, CUPB, BPN, WWB	213.17	129	1.65	0.94	0.94	0.07	0.06
Three-factor model: IUPB + CUPB, BPN, WWB	283.84	132	2.15	0.89	0.89	0.09	0.08
Two-factor model: IUPB + CUPB, BPN + WWB	288.82	134	2.16	0.88	0.89	0.09	0.08
Single-factor model: IUPB + CUPB + BPN + WWB	687.85	135	5.10	0.58	0.59	0.15	0.14

N = 207; IUPB = initiative UPB, CUPB = compulsory UPB, BPN = basic psychological needs satisfaction, WWB = workplace well-being; CFI = Comparative Fit Index; IFI = Incremental Fit Index; SRMR = Standardized Root Mean Square Residual; RMSEA = Root Mean Error of Approximation. Ideal model − fit indicators are: χ^2^ /df < 3, RMSEA < 0.08, CFI > 0.9, IFI > 0.9, SRMR < 0.08.

### Common method bias test

Given the self-reported nature of the data and the potential for common method bias (CMB) to inflate observed relationships^43^, we conducted a full collinearity test as recommended by^50^. This approach involves regressing each latent variable on all other latent variables in the model and examining the variance inflation factor (VIF) values. A VIF value exceeding 3.3 is considered indicative of pathological collinearity and hints at the presence of CMB^50^.

We performed the full collinearity test with SPSS 26.0. The latent variables included in the test were initiative UPB (IUPB), compulsory UPB (CUPB), autonomy satisfaction, competence satisfaction, relatedness satisfaction, and workplace well-being. For each latent variable, we calculated the VIF when it was regressed on all other latent variables. All VIF values were below the recommended threshold of 3.3, ranging from 1.27 to 2.56. These results indicate that common method bias does not pose a significant threat to the validity of our findings^50^.

### Descriptive statistics and correlations

Table 2 presents the means, standard deviations, correlations, and reliability coefficients for all variables. Key findings include:

**Table 2:** Means, standard deviations, and inter-correlations of all variables.

Variables	Mean±SD	1	2	3	4	5	6	7	8	9	10	11
Age	2.52±0.60											
Gender	1.55±0.50	0.01										
Education	2.95±0.56	0.18*	− 0.04									
Tenure	2.80±0.83	0.59**	− 0.05	0.31**								
Job position	2.04±0.84	0.47**	− 0.02	0.28**	0.54**							
IUPB	3.20±0.87	0.06	− 0.03	− 0.03	0.12	0.02						
CUPB	3.65±1.03	0.03	− 0.01	0.02	0.05	− 0.12	0.59**					
BPN	4.16±0.73	0.08	− 0.08	0.07	0.14*	0.30**	− 0.10	− 0.41**				
ANS	3.91±0.91	0.08	− 0.11	0.03	0.09	0.24**	− 0.17*	− 0.48**	0.84**			
RNS	4.05±0.92	0.02	0.01	0.04	0.10	0.24**	− 0.13	− 0.37**	0.86**	0.61**		
CNS	4.54±0.83	0.11	− 0.09	0.10	0.16*	0.27**	0.06	− 0.15*	0.76**	0.44**	0.47**	
WWB	4.54±0.79	0.02	− 0.08	0.05	0.03	0.22**	− 0.13	− 0.36**	0.75**	0.66**	0.64**	0.53**

N = 207; IUPB = initiative UPB, CUPB = compulsory UPB, BPN = Basic psychological needs satisfaction; ANS = autonomous need satisfaction, RNS = relatedness need satisfaction, CNS = competency need satisfaction, WWB = workplace well-being.

CUPB was significantly negatively correlated with basic psychological needs satisfaction (r = − 0.41, p < 0.01) and its subscales (autonomy: r = − 0.48, p < 0.01; relatedness: r = − 0.37, p < 0.01; competence: r = − 0.15, p < 0.05). CUPB was significantly negatively correlated with workplace well-being (r = -0.36, p < 0.01). IUPB was not significantly correlated with basic psychological needs satisfaction (r = − 0.10, p = 0.14) or workplace well-being (r = − 0.13, p = 0.06), except for a weak negative correlation with autonomy (r = − 0.17, p < 0.05). Basic psychological needs satisfaction was significantly positively correlated with workplace well-being (r = 0.52, p < 0.01), providing preliminary support for the mediating hypothesis.

### Hypothesis tests

#### Direct effects of UPB on workplace well-being

We tested the direct effects of IUPB and CUPB on workplace well-being using hierarchical regression analysis in SPSS 26.0 (IBM SPSS Statistics 26.0, Official URL: https://www.ibm.com/products/spss-statistics), controlling for age, gender, education level, organizational tenure, and job position. For IUPB, as shown in Model 10 of Table 3, after controlling for demographic variables, IUPB was not significantly related to workplace well-being (β = -0.13, p = 0.06). Thus, neither H1a nor H1b was supported. For CUPB, as shown in Model 10 of Table 3, CUPB was significantly negatively related to workplace well-being (β = − 0.33, p < 0.001), supporting H2.

**Table 3:** Multiple regressions results for initiative UPB.

Variables	BPN		ANS		RNS		CNS		Well-being	
	Mode 1	Model 2	Model 3	Model 4	Model 5	Model 6	Model 7	Model 8	Model 9	Model 10
Age	− 0.08	− 0.08	− 0.03	− 0.03	− 0.14	− 0.14	− 0.04	− 0.04	− 0.07	− 0.07
Gender	− 0.07	− 0.07	− 0.11	− 0.11	0.01	0.01	− 0.08	− 0.08	− 0.09	− 0.08
Education	− 0.02	− 0.03	− 0.04	− 0.05	− 0.03	− 0.04	0.03	0.03	− 0.01	− 0.01
Tenure	0.01	0.03	− 0.04	− 0.01	0.03	0.06	0.03	0.02	− 0.07	− 0.07
Job position	0.34***	0.34***	0.28**	0.27**	0.29	0.29**	0.27**	0.27**	0.30***	0.30***
IUPB		− 0.11		− 0.18**		− 0.13		0.05		-0.13

N = 207; IUPB = initiative UPB, BPN = Basic psychological needs satisfaction, ANS = autonomous need satisfaction, RNS = relatedness need satisfaction, CNS = competency need satisfaction, WWB = workplace well-being;

#### Effects of UPB on basic psychological needs satisfaction

We next tested the effects of IUPB and CUPB on basic psychological needs satisfaction (both overall and its subscales) using hierarchical regression, with demographic variables controlled for. For IUPB, as shown in Table 3, it was significantly negatively related to autonomy satisfaction (β = − 0.18, p < 0.01) but not to competence satisfaction (β = 0.05, p = 0.46), relatedness satisfaction (β = − 0.13, p = 0.07), or overall basic psychological needs satisfaction (β = − 0.11, p = 0.10), thus failing to support H3a. For CUPB, also as shown in Table 4, it was significantly negatively related to autonomy satisfaction (β = − 0.46, p < 0.001), relatedness satisfaction (β = − 0.34, p < 0.001), and overall basic psychological needs satisfaction (β = − 0.38, p < 0.001); however, its relationship with competence satisfaction was not significant (β = − 0.13, p = 0.07), thereby partially supporting H3b, as CUPB negatively predicted autonomy and relatedness satisfaction but not competence satisfaction.

**Table 4:** Multiple regressions results for compulsory UPB.

Variables	BPN		ANS		RNS		CNS		Well-being	
	Mode 1	Model 2	Model 3	Model 4	Model 5	Model 6	Model 7	Model 8	Model 9	Model 10
Age	− 0.08	− 0.08	− 0.03	− 0.02	− 0.14	− 0.13	− 0.04	− 0.04	− 0.06	− 0.06
Gender	− 0.07	− 0.07	− 0.11	− 0.11	0.01	0.01	− 0.08	− 0.08	− 0.08	− 0.08
Education	− 0.02	− 0.01	− 0.04	− 0.02	− 0.03	− 0.02	0.03	0.03	0.01	0.02
Tenure	0.01	0.07	− 0.04	0.03	0.03	0.08	0.03	0.05	− 0.10	− 0.05
Job position	0.34***	0.26**	0.28**	0.18*	0.29	0.22**	0.27**	0.24**	0.31***	0.23**
CUPB		− 0.38***		− 0.46***		− 0.34***		− 0.13		− 0.33***

N = 207; CUPB = compulsory UPB, BPN = Basic psychological needs satisfaction, ANS = autonomous need satisfaction, RNS = relatedness need satisfaction, CNS = competency need satisfaction, WWB = workplace well-being;

#### Mediating effects of basic psychological needs satisfaction

**Fig. 2:**
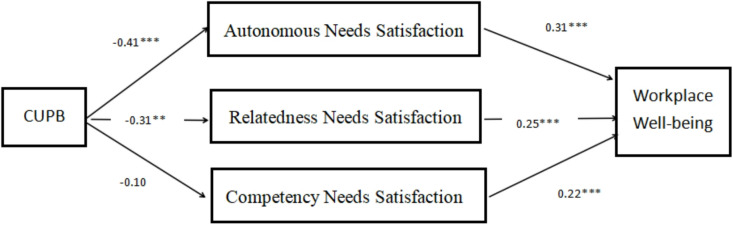
Mediation model of the effects of CUPB on workplace well-being via basic psychological needs satisfaction.

To test H4 (the mediating role of basic psychological needs satisfaction in the relationship between CUPB and workplace well-being), we used the PROCESS macro (Model 4)^51^, with 5,000 bootstrap samples to calculate 95% confidence intervals (CIs), where a mediation effect is considered significant if the CI does not include zero. As shown in Table 5, the total effect of CUPB on workplace well-being was significant (β = − 0.36, p < 0.001); regarding the indirect effects through the subscales of basic psychological needs, the indirect effect through autonomy satisfaction was significant (estimate = − 0.14, 95% CI = [− 0.21, − 0.08]), the indirect effect through relatedness satisfaction was significant (estimate = − 0.08, 95% CI = [− 0.13, − 0.04]), and the indirect effect through competence satisfaction was also significant (estimate = − 0.03, 95% CI = [− 0.06, − 0.01]). Additionally, after controlling for the mediating variables, the direct effect of CUPB on workplace well-being was no longer significant (β = -0.03, p = 0.43), indicating full mediation. These results thus support H4, confirming that the negative relationship between CUPB and workplace well-being is mediated by reduced satisfaction of autonomy, competence, and relatedness needs. The overall mediation model is presented in Fig. 2.

**Table 5:** Bootsrap analysis summary showing the indirect effect of CUPB on workplace well-being via basic psychological needs satisfaction.

Model pathways	Effect	BootSE	95% CI mean indirect effect (lower and upper)
Total effect	− 0.36	–	–
Direct effect	− 0.03	0.04	[− 0.11, 0.05]
Indirect effects			
a1b1:CUPB → ANS → WWB	− 0.14	0.03	[− 0.21, − 0.08]
a2b2:CUPB → RNS → WWB	− 0.08	0.02	[− 0.13, − 0.04]
a3b3:CUPB → CNS → WWB	− 0.03	0.01	[− 0.06, − 0.01]
Contrasts of indirect effects			
Autonomy vs. Relatedness	− 0.05	0.04	[− 0.14, 0.03]
Autonomy vs. Competence	− 0.11	0.03	[− 0.18, − 0.05]
Relatedness vs. Competence	− 0.05	0.02	[− 0.11, − 0.01]

N = 207; ANS = autonomous need satisfaction, RNS = relatedness need satisfaction, CNS = competency need satisfaction, WWB = workplace well-being.

Additionally, we compared the magnitude of the mediating effects using the contrast function in PROCESS. The mediating effect of autonomy satisfaction (estimate = − 0.14) was larger than that of relatedness (estimate = − 0.08) and competence (estimate =− 0.03). There was no significant difference between autonomy and relatedness (contrast = − 0.05, 95% CI = [− 0.14, 0.03]), but autonomy had a significantly larger effect than competence (contrast = − 0.11, 95%CI = [− 0.18, − 0.05]), and relatedness had a significantly larger effect than competence (contrast = -0.05, 95%CI = [− 0.11, − 0.01]).

## Discussion

This study set out to address the unresolved debate regarding the relationship between unethical pro-organizational behavior (UPB) and employees’ workplace well-being by distinguishing between two subtypes of UPB—initiative UPB (IUPB) and compulsory UPB (CUPB)—and examining the mediating role of basic psychological needs satisfaction derived from self-determination theory (SDT). The findings reveal three key insights. First, UPB is indeed multidimensional, with IUPB and CUPB demonstrating distinct patterns of association with workplace well-being. Second, CUPB negatively affects workplace well-being, whereas IUPB shows no significant direct effect. Third, basic psychological needs satisfaction fully mediates the negative relationship between CUPB and workplace well-being, with autonomy, competence, and relatedness exhibiting differential mediating magnitudes. These findings contribute to the literature in several meaningful ways.

A central contribution of our study is that it empirically demonstrates the multidimensional nature of UPB. Although prior research has introduced the conceptual distinction between IUPB and CUPB, most empirical studies have continued to conceptualize and operationalize UPB as a homogeneous construct, assuming that all forms of UPB have similar psychological consequences^13^^.^ Our findings challenge this assumption and demonstrate that the two subtypes of UPB, although with moderate correlations (r = 0.59), actually have different effects on workplace well-being. This finding corroborates recently-advanced calls in the literature on UPB to move beyond unidimensional conceptions of UPB^2,3^ and highlights that failing to differentiate between voluntary and compelled unethical behaviors hides away the subtle ways in which UPB influences employee outcomes.. By providing empirical evidence for the distinctiveness of IUPB and CUPB, our study paves the way for further research to uncover antecedents and consequences of each type of prototype separately.

These mixed findings in prior UPB research, reporting harmful consequences such as guilt, shame, and work-to-life conflict^9,11^ or reporting neutral and even positive effects such as greater self-esteem and positive emotions^3,12^, have puzzled organizational behavior scholars for a long time. Our results suggest why this inconsistency exits: incongruous motivational components (IUPB) and congruous motivational components (CUPB) of UPB appear to have been inadvertently mixed across prior studies. Examining CUPB and IUPB separately, we found that CUPB consistently impoverished well-being (β = − 0.33, p < 0.001), whereas IUPB showed no significant association (β = − 0.13, p = 0.06), implying that the psychological cost of UPB is not rooted in the behavior itself but follows from the context of motivation in which it is embodied. Specifically, when an actors’ unethical behavior is perceived as compelled by external pressures, it is psychologically costly; when their unethical behavior is voluntary, their behaviors may be offset by countervailing forces^10^. By clarifying this contingency, our study offers a theoretical lens to reconcile seemingly contradictory findings and suggests that future research needs to consider voluntariness as a boundary condition.

Our second major contribution lies in identifying basic psychological needs satisfaction as the mechanism linking CUPB to impaired well-being. Prior research has predominantly invoked conservation of resources (COR) theory to explain the consequences of UPB^11,12^, focusing on resource depletion as the core mechanism behind this explanation. While COR theory provides a useful starting point, it does not fully specify why compelled unethical behavior should be particularly damaging compared to voluntary unethical behavior. By integrating SDT, we illustrate that CUPB frustrates basic needs for autonomy (β = − 0.46, p < 0.001), competence (β = − 0.13, p = 0.07, non-significant), and relatedness (β = − 0.34, p < 0.001), thereby decreasing workplace well-being. Importantly, the full mediation model (indirect effects mediated through autonomy: − 0.14, 95% CI [− 0.21, − 0.08]; relatedness: − 0.08, 95% CI [− 0.13, − 0.04]; competence: − 0.03, 95% CI [− 0.06, − 0.01]) confirm that need frustration, not resource depletion, accounts for the negative effect of CUPB. These findings go beyond the applicability of SDT to the domain of unethical behavior and show how behaviours supposedly undertaken to benefit the organization can produce either positive or negative psychological effects, depending on the motivational quality (autonomous or controlled) of the behaviour.

An additional nuance revealed by our analyses is that the three basic psychological needs do not mediate the CUPB–well-being relationship equally. The strongest mediator was autonomy satisfaction (indirect effect = − 0.14), followed by relatedness (indirect effect = − 0.08) and then competence (indirect effect = − 0.03). The contrast analyses confirmed that autonomy’s mediating effect was significantly larger than competence’s (contrast = − 0.11, 95% CI [− 0.18, − 0.05]), and relatedness’s effect was significantly greater than competence’s (contrast = − 0.05, 95% CI [− 0.11, − 0.01]). This pattern is consistent with SDT’s emphasis on volition as the engine of psychological functioning^31^ and shows that the loss of perceived control characteristic of CUPB is the primary pathway through which it has a deleterious effect on well-being. At the same time, the significant mediation through relatedness reveals that CUPB also harms well-being by damaging social connections—employees might feel alienated from others who disapprove of their actions or resent supervisors who pushed them to do it. The weaker mediation through competence suggests that although CUPB may result in self-doubt, it does so less strongly than it affects autonomy and relatedness. This result corroborated prior research that shows although all three needs are essential, each makes different contributions to well-being^52,53^ and highlights the need to consider each of them individually, as distinct phenomena rather than pooling them into a single measure. This complexity has implications for intervention design: efforts to ameliorate the negative impact of CUPB should especially stress the reconstruction of autonomy and repair of social connections.

## Practical implications

Our study thus offers concrete, evidence-based implications for organizations and managers. Each of our recommendation is evidence-based and anchored in specific findings of our analyses.

First, organizations should push to reduce compulsory UPB by redesigning their performance management systems. Our findings indicate that CUPB had a significant negative direct effect on workplace well-being (β = − 0.33, p < 0.001), and this effect operates via the frustration of basic psychological needs. Managers should interrogate whether their expectations of performance are inadvertently making it necessary for employees to engage in unethical behaviour. For example, to set an unattainable sales quota or to suggest, “results above all else”, creates an environment in which employees might see themselves pressured to misrepresent information to attain them. In their place, organizations should adopt approaches such as the balanced scorecard approach, which should include ethical conduct as one of its goals. Concrete action steps include: (a) auditing frequently to see when employees say they feel pressured to bend rules. (b) Setting performance targets at a level they can be met without bending rules. (c) Keeping managers accountable not just for team performance but also for the culture they develop.

Second, managers should proactively support their employees’ basic psychological needs (especially related to autonomy and relatedness). Our mediation analyses showed that autonomy satisfaction (indirect effect = − 0.14) and relatedness satisfaction (indirect effect = − 0.08) were the main effects by which CUPB decreased well-being. To build up employees’ autonomy, managers can provide employees with more autonomy over how they work to meet targets, incorporate employees in target-setting activities, and tell employees clearly that they can safely refuse actions related to ethics without fear of negative consequences. To enhance employees’ relatedness, managers need to create a team environment in which employees can safely express concern about ethics, reward employees who act ethically even under pressure, and ensure that employees’ supervisors offer them help emotionally when they come under ethical pressure. Importantly, these interventions are not only ethically but also empirically validated: our data show that when basic psychological needs are satisfied, workplace well-being increases significantly (r = 0.52, p < 0.01).

Third, organizations should provide structured, ethics-based decision-making training to equip employees to resist unethical pressures. Our findings indicated that CUPB was particularly damaging because it was externally compelled. In an ethics-based decision-making training programme, employees can be trained to recognize themselves when they are being pressured into acting unethically, convey their concerns to their superior, and learn of alternatives to managing the goals of their organization in ways that do not include acting unethically. For example, ethics-based decision-making workshops can raise common sales scenarios in which UPB may occur (for example, being asked to hide negative information from a client), providing employees with scripts and decisions to take in a certain way that employees can use ethically. Such training should be supplemented by company policies designed to assure employees being subjected to an unethical pressure report that they will not be punished. By training employees to resist CUPB, organizations protect workplace well-being, while protecting organizational ethics.

## Limitations and future directions

Our study has several limitations that should be considered in future research. Instead of listing limitations, we discuss how each limitation might suggest future directions for research.

First, with regard to causal inference, the three-wave cross-sectional design limits our ability to infer causality. Although the temporal order of the variables (T1: UPB subtypes; T2: basic psychological needs; T3: workplace well-being) fits the hypothesized order of causality, we cannot necessarily rule out reverse causality, or unmeasured third variables. Future work could consider experimental or longitudinal designs—experimentally manipulating IUPB versus CUPB via scenarios in experimental laboratory settings or experience-sampling designs to capture within-person variability of experiences over time—to establish these causal pathways.

Second, our sample is limited to Chinese salespeople and therefore limits the generalizability of our findings. Because sales jobs are typically high-pressure jobs that involve frequent interactions spanning boundaries, sales is an apt context in which to test UPB. However, it is unclear whether our findings would generalize to other jobs (e.g., health care, education, manufacturing) or to other cultures (e.g., those in Western countries with different cultural norms and regulatory environments) as well. Future studies should replicate this research with samples drawn from different jobs, different industries, and different cultures, and examine the cross-cultural validity of our proposed model. Of particular interest for this are cross-cultural comparisons in which one could test whether the distinction between IUPB and CUPB is equally salient in collectivist cultures versus individualist cultures.

Third, the non-significant association between IUPB and workplace well-being was unexpected. While our findings suggest that IUPB does not significantly harm well-being, this null effect may mask important heterogeneity. Future investigations could explore potential moderating variables that might shape this relationship. For example, IUPB might improve well-being in workplaces with a strong ethical climate where such behaviour is seen as legitimate (i.e., going “above and beyond” within limits of ethical principles), but it may undermine well-being in workplace climates where ethical standards are lax and where employees later experience feelings of moral distress. Or individual level differences, such as moral identity, level of ethical ideology, may moderate whether IUPB is perceived as positive or negative. Exploring these moderators would provide further insight as to when and for whom IUPB matters.

Fourth, even though our study explores basic psychological needs as mediators, there may be other pathways that explain them. Although SDT proved to be a powerful explanatory lens for CUPB effects, other pathways, such as emotional labour, cognitive dissonance, or moral disengagement, could also connect UPB to well-being. For example, employees who engage in CUPB may experience emotional labour because they are experiencing the affective burden of the incongruity between their internal and external states, a state that dissipates personal energy independently of need frustration. Future research could bridge these explanations to construct a fuller picture of the UPB psychological consequences.

Finally, our measure of UPB was based on self-reports, which may be subject to social desirability bias. We assured anonymity and collected data over 3 waves to address this issue, but in the future, peer and supervisor ratings of UPB could be used to validate UPB in multiple sources. Alternatively, investigators could use measures of UPB that are indirectly measured, such as a scenario test or an implicit association test, to capture UPB traits, but in a manner that is far less susceptible to social desirability bias.

## Conclusion

Our study contributes to the literature on unethical pro-organizational behaviour (UPB) by differentiating between initiative unethical pro-organizational behaviour (IUPB) and compulsory unethical pro-organizational behaviour (CUPB) and considering their differential consequences on employees’ workplace well-being. Drawing on self-determination theory, our research highlights the role of frustration of basic psychological needs: autonomy, competence, and relatedness—as a key partial explanation for the harmful consequences of CUPB. The findings of our study imply several key conclusions. First, not all forms of UPB are harmful: CUPB harms workplace well-being, whereas IUPB does not have a significant direct effect. Second, the negative consequences of CUPB are completely mediated by frustration of basic psychological needs with autonomy as the strongest mediating factor, followed by relatedness and competence. Third, motivational context, whether behaviour is voluntary or forced externally, determines the psychological consequences of UPB, providing useful clarification of mixed evidence reported in earlier work.

Theoretically, this study advances UPB research by confirming UPB’s multidimensional structure, grounding self-determination theory in the realm of unethical behavior, and elucidating why and how compelled unethical acts undermine employee well-being. Practically, this study tells organizations the need to alleviate coercive pressures in the workplace, cultivate an ethical climate, and support employees’ basic psychological needs to protect their well-being. In sum, this study underscores the importance of differentiating the UPB subtypes and, at the same time, underscores motivational and psychological processes that serve to shape employee outcomes. By targeting antecedents of and harms of CUPB, organizations can cultivate ethical behavior, support employee well-being, and build healthier and sustainable work environments.

## Data Availability

The datasets used and/or analyzed during the current study are available from the corresponding author upon reasonable request.
